# The Link between Fibromyalgia Syndrome and Anger: A Systematic Review Revealing Research Gaps

**DOI:** 10.3390/jcm11030844

**Published:** 2022-02-05

**Authors:** Carmen M. Galvez-Sánchez, Gustavo A. Reyes del Paso, Stefan Duschek, Casandra I. Montoro

**Affiliations:** 1 Department of Psychology, Faculty of Humanities and Educational Sciences, University of Jaén, 23071 Jaén, Spain; greyes@ujaen.es; 2 Department of Psychology, Institute of Psychology, UMIT-University for Health Sciences, Medical Informatics and Technology, 6060 Hall in Tyrol, Austria; stefan.duschek@umit.at

**Keywords:** fibromyalgia syndrome, anger, pain, intervention, health-related quality of life

## Abstract

Anger has been associated with increased pain perception, but its specific connection with Fibromyalgia Syndrome (FMS) has not yet been established in an integrated approach. Therefore, the present systematic review focuses on exploring this connection, and based on this connection, delimiting possible gaps in the research, altogether aimed at improving FMS clinical intervention and guiding future research lines. Anger is considered a basic negative emotion that can be divided into two dimensions: anger-in (the tendency to repress anger when it is experienced) and anger-out (the leaning to express anger through verbal or physical means). The current systematic review was performed based on the guidelines of the PRISMA and Cochrane Collaborations. The Prospective Register of Systematic Reviews (PROSPERO) international database was forehand used to register the review protocol. The quality of chosen articles was assessed and the main limitations and research gaps resulting from each scientific article were discussed. The search included PubMed, Scopus, and Web of Science databases. The literature search identified 13 studies eligible for the systematic review. Levels of anger-in have been shown to be higher in FMS patients compared to healthy participants, as well as patients suffering from other pain conditions (e.g., rheumatoid arthritis). FMS patients had also showed higher levels of state and trait anxiety, worry and angry rumination than other chronic pain patients. Anger seems to amplify pain especially in women regardless FMS condition but with a particularly greater health-related quality of life´s impact in FMS patients. In spite of the relevance of emotions in the treatment of chronic pain, including FMS, only two studies have proposed intervention programs focus on anger treatment. These two studies have observed a positive reduction in anger levels through mindfulness and a strength training program. In conclusion, anger might be a meaningful therapeutic target in the attenuation of pain sensitivity, and the improvement of the general treatment effects and health-related quality of life in FMS patients. More intervention programs directed to reduce anger and contribute to improve well-being in FMS patients are needed.

## 1. Introduction

There is controversy on the conceptualization of anger [1]. Although there exists a theoretical mismatch about the nature and definition of anger, its complex and dynamic conceptualization is recognized [2,3,4]. In this sense, anger is considered one of the basic emotions together with fear, disgust, sadness, happiness, and surprise [1,4,5]. Anger may be both, a state and a personality trait [1,6]. Furthermore, anger is mainly expressed in two basic dimensions: anger towards others or outside (also called anger-out), and anger towards oneself or within (which is also known as anger-in, anger turned inward, or unexpressed anger). On purpose, these two basic dimensions have been deeply studied in relation to anger management (i.e., as anger-in (the tendency to repress anger when it is experienced) and anger-out (the leaning to express anger through verbal or physical forms)) [7], and the sensitivity to both, acute and chronic pain [8].

Related to the neurological correlates of anger, several brain structures have been involved in both anger and aggression, including those related with emotion regulation (i.e., the amygdala) [1,5]. For instance, studies focus on human brain imaging have reported greater activity in anterior cingulate cortex and orbitofrontal cortex when individuals are asked to recall past experiences that made them feel angry [5,9]. Historically, studies of anger and aggression have been conducted to explore the involvement of subcortical structures in emotion [1]. Among these structures, the hypothalamus has been one of the earliest and leading associated to anger and aggressive behavior, and based on it a neural circuit for anger and aggression has been frequently proposed [1].

In addition, the neurotransmitter serotonin has also been proposed to play a relevant role in the regulation of anger and aggression [1,9]. In fact, the serotonin deficiency hypothesis (that is, the causal role of diminished serotonin in anger and aggression) is widely supported by the scientific evidence. This evidence shows clearly that aggression is inversely related to serotonergic activity [1,5].

Chronic pain patients usually experience anger [10,11]. Nonetheless, it tends to be underestimated due to the denial and negative social connotation of this emotion [12], especially in women [13]. A possible explanation for anger denial can be related to the social norms and religious values which are assumed to repress its expression [14]. Chronic pain patients, compared to healthy controls, have frequently reported higher levels of anger suppression and/or hostility, which in turn have been related to increased pain and disability [10,11,15]. Kerns et al. [16] reported that the internalization of angry feelings explained a considerable part of the variance in pain intensity, perceived interference and reported frequency of pain behaviors in chronic pain patients. Gaskin et al. [17] revealed that state anger may be a significant predictor of the affective pain. Anger-in has also significantly been associated with depression and pain, whereas anger-out has been linked to greater disability in chronic pain patients [10].

One of the prototypical chronic pain conditions is fibromyalgia syndrome (FMS), which is characterized by generalized and persistent non-inflammatory musculoskeletal pain. Accompanying symptoms frequently comprise depression, anxiety, fatigue, insomnia, morning stiffness, and cognitive impairments (i.e., memory and attention problems, concentration difficulties, mental slowness, etc.) [18,19]. Moreover, a high percentage of FMS patients generally exhibited negative affect, which encompass alexithymia, catastrophizing, neuroticism [20,21,22,23] and deficit in health-related quality of life [21,22,24]. Its prevalence is stablished at 2 to 4% in the general population, and seems to be more frequent in women than in men [18]. However, recent studies reveal a possible gender bias which leads healthcare professionals to overestimate FMS prevalence in women simultaneously is underestimated in men [25,26]. Nowadays, there is not a specific treatment for FMS. Therefore, research should contribute to the clinical practice.

Regarding the empirical investigation of the role of anger in FMS, the available literature is scarce. Several authors have pointed out that FMS patients tend to experience higher levels of anger, in comparison with rheumatoid arthritis (RA) patients and healthy controls [27,28,29]. In the same line, RA patients are considered to be more prone to experience anger (trait anger) in addition to readily express it, while anger is rather internalized and suppressed by FMS patients [30]. Inhibited anger has been linked to greater intense pain than uninhibited anger [31,32], this being, in fact, more common in FMS patients [20,27]. On this detail, physiological arousal has been proposed as a mechanism that underlies the effect of anger in pain intensity due to effortful suppression, and the counterintuitive rise of the accessibility of angry thoughts and feelings after suppression [7,33]. Additionally, both enhanced [27,34] and reduced [35,36] pain has been reported after anger expression (measured by the ‘Anger Expression Inventory’, experimental emotion-induction tasks, etc.) [34,35]. These mixed findings may be explained by the trait-by-state matching model [37]. This model proposes a reduction of anger and its negative consequences such as pain by the matching of a general style to express anger with actual anger-expressive behavior [36]. Oppositely, according to the same model, the lack of anger expression in high trait anger-out individuals intensifies anger and pain [36]. Furthermore, higher pain and anger has been linked to poorer health and quality of life in FMS [24].

Due to the fact that social constraints discourage the expression of anger, overall, in women [13], frequent mismatches may arise between the urge to express anger and its actual expression [38]. Furthermore, given that anger is associated with negative clinical outcomes in FMS, it can be argued that its addressing in clinical practice might be beneficial for optimizing treatment and improving health-related quality of life of these patients. However, fewer studies exploring the beneficial effects of its intervention have been conducted. Based on the above reviewed literature, the present systematic review focuses on exploring the connection between anger and FMS and, based on it, delimiting possible gaps in the research, altogether aimed at improving FMS clinical intervention and guiding future research on this topic.

## 2. Materials and Methods

### 2.1. Search Strategy

Accordingly, to the guidelines of the Cochrane Collaboration and the Preferred Reporting Items for Systematic Reviews and Meta-Analyses (PRISMA), the current systematic review was conducted [39]. The Prospective Register of Systematic Reviews (PROSPERO) international database was forehand used to register the review protocol (registration ID: CRD42021226843). The search terms included: “fibromyalgia” and “anger”. The terms were extracted from the Medical Subject Headings (MeSH). The PICO question was: Which is the relation between fibromyalgia and anger and the possible gaps on the -based on it- lines of research?

**Figure 1:**
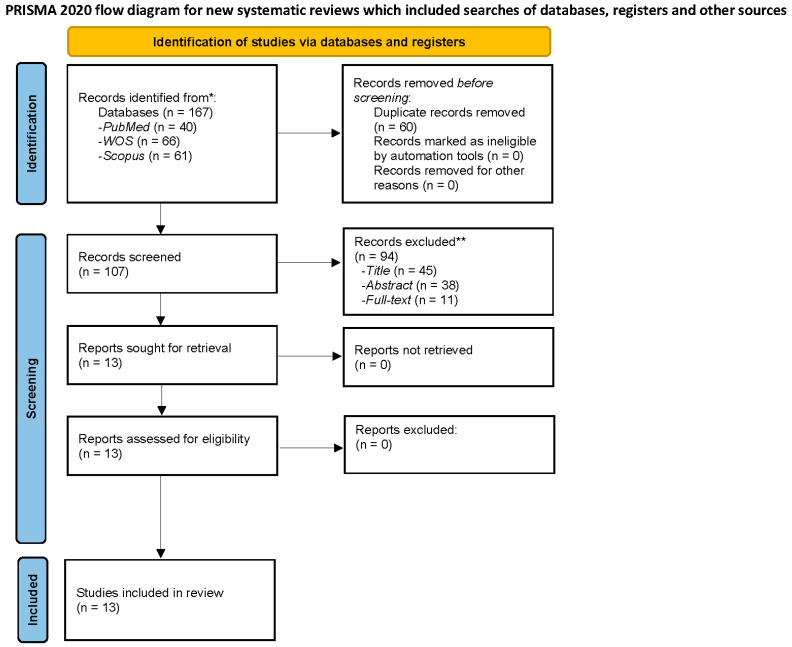
Flow diagram of FMS and anger.

Two researchers (C.M.G.-S. and C.I.M.) independently searched the PubMed, Scopus, and Web of Science (WOS) databases. In case of exist any discrepancy, it was solved by consensus between authors. All articles were independently screened by the two researchers previously mentioned (C.M.G.-S. and C.I.M.), who also selected the studies which fulfill the inclusion criteria for the subsequent full text analysis. In addition, the titles and abstracts of the articles were revised to eliminate irrelevant research according to the review objectives; later, the selected articles were analyzed in depth. In accordance with the inclusion and exclusion criteria, the full texts of relevant articles were screened to reach a final set of articles to be included in the revision. C.M.G.-S. and C.I.M. decided whether to include or exclude the different studies that arose in the preliminary screened and any discrepancies were solved by all authors, who made the final judgement regarding the inclusion of each research. In the PRISMA flowchart (Figure 1) the screening and selection processes are shown for better comprehension. Before data extraction and quality assessment, C.M.G.-S. and C.I.M. revised the final set of articles to verify their eligibility for this systematic review. The last search was performed on 26 November 2021.

### 2.2. Eligibility Criteria

The inclusion criteria comprise: (1) peer-reviewed original studies of FMS and anger, (2) adult patients (≥18 years old) with an official diagnosis of FMS (American College of Rheumatology official criteria); and (3) English-written articles. Likewise, articles were excluded if they were (1) review article or meta-analysis; (2) comment, editorial, case report, letter, or meeting/congress abstract; and (3) non-English publication.

### 2.3. Data Extraction and Quality Assessment

C.M.G.-S. and C.I.M., independently extracted the characteristic, methodologies and main results of each article; solving any discrepancies between all authors. To elaborate the Table 1, the following data was retrieved: first author, study name, country, year of publication, study design, sample size (participant age and sex), total of participants in each study group, and the diagnostic criteria of FMS. The studies details can be consulted in Table 1. The other two authors (G.A.R.d.P. and S.D.) reviewed all the data to guarantee the quality and precision of the extraction.

With the objective to assess the quality of the selected articles, both C.M.G.-S. and C.I.M. independently assessed and explored the limitations of each study. As in previous occasions, any discrepancies in the analysis of limitations were discussed by all authors, who made the final decision together.

### 2.4. Data Synthesis

Considering the purpose of this systematic review, the authors checked the main objectives of each study, the methodology, and if there were or not healthy control groups included. The clinical relevance of the main findings and the principal limitations of each research were also determined (See Table 1 for more detail). The analysis of the characteristics of each study and their quality and limitations—following the guidelines of the Cochrane Collaboration and the Preferred Reporting Items for Systematic Reviews and Meta-Analyses (PRISMA)—were made to better knowledge of the connection between FMS and anger and to stablish possible research gaps; the last with the objective to improve FMS clinical intervention and guide future research lines.

## 3. Results

### 3.1. Literature Search and Study Characteristics

A total of 167 articles were identified among all database searches, and 107 were finally chosen for screening. The detailed inclusion process may be reviewed in the PRISMA flow chart (Figure 1). Finally, 24 full-text articles were analyzed to assess their eligibility for the current systematic review. Only 13 articles fulfilled the inclusion criteria, therefore, they were included in the data extraction (Table 1) and quality assessment processes. 

The selected studies were published between 2000 and 2019. Within the 13 studies, 11 were cross-sectional [20,24,27,28,29,30,40,41,42,43,44,45], 1 longitudinal [46], and 1 was a clinical trial [47]. Of the total of selected studies, nine studies were conducted in Europe [24,27,29,40,41,42,44,45,46], one in the United States of America [28], two in Brazil [43,47] and one in Israel [30]. The 13 selected studies include a total of 1511 participants, of which 902 are women with FMS (age range: 40–58 years old) and 595 women belong to the control groups: 151 women with another chronic pain disorder: 71 RA patients (age range: 45–46 years old), 50 chronic low back pain patients (age mean: 47 years old), 30 osteoporosis and/or osteoarthritis patients (age mean: 59 years old) and 444 healthy women (age range: 38–52 years). In total, only 14 men (9 men with FMS and 5 healthy men) participated particularly in one of the studies [24]. 

**Table 1:** Characteristics of relevant eligible studies regarding FMS and Anger.

First Author (Publication Year), Study Name, Country	Objective	Study Design	Sample Size [Mean ± Age (SD)]	FMS Diagnostic Criteria	Instruments	Variables and Results
Amir et al., 2000 [30]. Coping styles, anger, social support, and suicide risk of women with Fibromyalgia Syndrome. Israel.	To examine some personal dispositions in FMS patients.	Cross-sectional.	N = 220 female participants. 51 FMS patients (48.96 ± 8.41). 51 RA patients (46.25 ± 13.61). 50 CLBP patients (47.12 ± 11.61). 50 HW (45.66 ± 13.11).	1990 ACR.	Coping Inventory for Stressful Situations. STAXI. Suicide Risk Scale. Social Support Scale.	CP patients: similar personality traits. High coping style of avoidance and anger (especially: state anger and anger-in). FMS patients: not significant differences from the other patient groups on any variables. Not have a characteristic personality pattern.
Sayar et al., 2004 [27]. Alexithymia and anger in patients with Fibromyalgia. Turkey.	To delineate the relevance of the personality construct alexithymia and anger-in in patients with FMS.	Cross-sectional.	N = 112 female participants. 50 FMS patients (40.50 ± 8.80). 20 RA patients (45.60 ± 14.90). 42 HW (38.80 ± 10.40).	1990 ACR.	FIQ. BDI. BAI. STAXI. VAS. TAS.	FMS patients: higher anger-in than in RA patients. Anger-out and anxiety predicted the level of pain severity. In spite of anger-in is higher in FMS, it is the behavioral expression of anger, together with anxiety, that predicts the pain severity.
Shelley-Tremblay et al., 2009 [28]. The effects of sucrose consumption on left-frontal asymmetry and anger in persons with Fibromyalgia Syndrome. United States of America.	To determine whether FMS patients differ from age-matched healthy normal controls in their reaction to 75 g of sucrose by measuring the time course of self-report and electrophysiological responses.	Cross-sectional.	N = 18 female participants. 8 FMS patients (48.05 ± 6.14). 10 HW (47.05 ± 7.08).	1990 ACR.	Demographic Questionnaire. FIQ. HADS. POMS. Carbohydrate Addict’s Scale. Sucrose test meal beverage. Apparatus: Biopac MP30. Scan 4.2. An Accu-Chek Advantage.	FMS patients: higher levels of depression, anger, and other indicators of distress at all time points and increased rLFA than HW. Correlation between anger an increased rLFA.
Van Middendorp et al., 2010 [44]. The effects of anger and sadness on clinical pain reports and experimentally-induced pain thresholds in women with and without fibromyalgia. Netherlands.	To examine the effects of experimentally induced anger and sadness on self-reported clinical and experimentally-induced pain in a sample of women with and without fibromyalgia.	Cross-sectional.	N = 121 female participants. 62 FMS patients (46.30 ± 10.80). 59 HW (48.90 ± 11.40).	1990 ACR.	PANAS-X. VAS. Emotion induction procedure: autobiographical recall procedure. Experimentally-induced pain (electrical pain induction) measures: Sensory threshold. Pain threshold. Pain tolerance.	FMS patients: anger and sadness amplify pain in women with and without FMS. A stronger emotion-induced pain response was associated with more emotional reactivity. Anger and sadness reactivity to the emotion inductions were associated with greater increases in clinical pain responses. No convincing evidence was found for a larger sensitivity to anger and sadness in women with FMS than in women without FMS, or for a larger sensitivity to anger than to sadness in FMS.
Van Middendorp et al., 2010 [29]. Effects of anger and anger regulation styles on pain in daily life of women with fibromyalgia: a diary study. Netherlands.	To examine, among patients with fibromyalgia, whether anger during everyday life amplifies pain and whether general and situational anger inhibition and anger expression modulate the anger–pain link.	Cross-sectional.	N = 333 female participants. 333 FMS patients (47.00 ± 12.01).	1990 ACR.	Diary. SECS. Daily anger questions. VAS.	FMS patients: state anger predicted higher end-of-day pain in half of the patients, but lower pain in one-quarter of patients. State anger inhibition was unrelated to pain. Trait anger inhibition was related to more pain. Lowest pain level among patients with high trait anger expression who actually expressed their anger in an anger-arousing situation.
González-Roldán et al., 2013 [41]. Altered psychophysiological responses to the view of others’ pain and anger faces in fibromyalgia patients. Spain.	To examined brain activity, corrugator muscle electromyography (EMG), and heart rate (HR) responses to others’ faces expressing pain in patients with fibromyalgia.	Cross-sectional.	N = 40 female participants. 20 FMS patients (53.40 ± 8.10). 20 HW (52.70 ± 9.90).	1990 ACR.	Semistandardized interview. BDI. STAI. PANAS. EHI. WHYMPI (only in FMS). Emotional Face Task. Psychophysiological Recordings: Corrugator EMG activity, HR, and EEG signals.	FMS patients: greater cardiac deceleration to all facial expressions than pain-free controls, and enhanced N100 amplitudes to pain and anger faces in comparison with neutral faces. Greater theta power in response to pain and anger faces, as well as more reduced alpha power than pain-free controls to all faces.
Amutio et al., 2015 [46]. Mindfulness training for reducing anger, anxiety, and depression in Fibromyalgia patients. Spain.	To verify whether the application of a mindfulness-based training program was effective in modifying anger, anxiety, and depression levels in a group of women diagnosed with FMS.	Longitudinal Study.	N = 32 female patients. 32 FMS patients (51.82 ± 10.18): 14 experimental group and 18 control group (waiting list).		BDI. STAI. STAXI-2. Mindfulness intervention program.	FMS patients: a significant reduction of anger (trait) levels, internal expression of anger, state anxiety, and depression as well as a significant increase in internal control of anger. Mindfulness-based treatment was effective after 7 weeks. Results were maintained 3 months after the end of the intervention.
Ricci et al., 2016 [42]. Worry and anger rumination in Fibromyalgia Syndrome. Italy.	(1) To investigate the psychological profile of patients with FMS as compared to patients with other chronic pain syndromes (CP) and healthy subjects (HS) and (2) To examine the associations between anxiety, depression, worry and angry rumination in FMS patients.	Cross-sectional.	N = 90 female participants. 30 FMS patients (54.00 ± 12.00). 30 (59.00 ± 13.00) women with other type of CP (osteoporosis and osteoarthritis). 30 HW (age not specified).	1990 and 2010 ACR.	Socio-demographic information form. STAI. PSWQ. BDI-I. ARS.	FMS patients: higher levels of state and trait anxiety, worry and angry rumination than CP patients and HS. Worry and angry rumination were strongly associated in FMS.
Di Tella et al., 2017 [40]. Alexithymia, not fibromyalgia, predicts the attribution of pain to anger-related facial expressions. Italy.	To test the hypothesis that the attribution of pain to emotional facial expressions (other than pain) is greater in patients with FMS.	Cross-sectional.	N = 123 female participants. 41 FMS patients (50.80 ± 10.20). 82 HW (51.70 ± 8.40).	Expert rheumatologist.	HADS. TAS-20. Emotional Pain Estimation and Emotional Pain Ascription Task: modified version of the Ekman 60 Faces Test.	FMS patients: not increased attribution of pain to facial expressions of emotions. Alexithymic individuals demonstrated no specific problem in the recognition of basic emotions, but attributed significantly more pain to angry facial expression.
Offenbaecher et al., 2017 [24]. Struggling with adversities of life: the role of forgiveness in patients suffering from Fibromyalgia. Germany.	(1) To compare the magnitude and direction of associations between forgiveness and pain, mental and physical health, quality of life, and anger in a sample of FMS participants and healthy controls, and (2) To compare FMS and controls on mean levels of these variables.	Cross-sectional	N = 254 participants. 173 FMS patients (161 women and 9 men) (58.00 ± 8.80). 81 HP (76 women and 5 men) (47.20 ± 14.20).	Not specified.	Initial survey. Demographic questions: age, education, religion, sex and marital status. Two questions about the degree of religiosity and spirituality. Forgiveness of self and others scales. HADS. SF-12. SF-16. STAI-II. VAS. Regional pain scale.	FMS patients: higher pain and anger and poorer health and quality of life. Lower levels of both forgiveness of self and others.
El Tassa et al., 2018 [43]. Mood states, depressive symptoms, and physical function in women with Fibromyalgia. Brazil.	To investigate the relationship between mood states, depressive symptoms, and physical performance in women with FMS.	Cross-sectional case-control study.	N = 45 female participants. 28 FMS patients (44.80 ± 5.50). 17 HW (43.40 ± 4.70).	ACR 1990.	BRUMS. BDI. HAQ. VAS. Threshold painful sensibility: dial algometer. Physical Function: 6-min Walk Test; Sit and Reach Test; 8-ft Up Go Test; and 30-s Chair Stand Test. Knee flexion and extension maximum isometric voluntary contractions (MIVC).	FMS patients: tension and anger showed a positive correlation with tests that demand strength in knee extension.
Andrade et al., 2019 [47]. Acute effect of strength training on mood of patients with fibromyalgia syndrome. Brazil.	To analyze the acute effect of strength training (ST) sessions on the mood states of patients with fibromyalgia.	Clinical trial.	N = 28 female participants. 28 FMS patients (51.88 ± 10.22).	1990 and 2016 ACR.	Sociodemographic and clinical data: self-reported instrument. BRUMS. Strength training program.	FMS patients: the ST practice had positive effects on the patients’ mood states after a single session. Reductions in anger, mental confusion, mood depression, fatigue, and tension, due to ST program.
Toussaint et al., 2019 [45]. Anger rumination mediates differences between fibromyalgia patients and healthy controls on mental health and quality of life. Germany.	To examine differences between fibromyalgia patients and healthy controls on anger rumination, mental health and quality of life and tested anger rumination as a mediator of patient-control differences in mental health and quality of life.	Cross-sectional.	N = 116 female participants. 58 FMS patients (58.80 ± 8.80). 58 HW (47.00 ± 14.20).	Having an FMS diagnosis (any criteria specified).	Socio-demographics data: age, sex, and educational level. ARS. HADS. SF-12. SF-16.	FMS patients: higher anger rumination scales and depression and anxiety and lower on quality of life. All anger rumination scales were related to poorer mental health and quality of life. Patient–control differences on mental health and quality of life were mediated by anger rumination. The only subscale with mediating effects was anger memories.

Abbreviations: ACR: American College of Rheumatology’s Criteria; ARS: Anger Rumination Scale; BAI: Beck Anxiety Inventory; BDI: Beck Depression Inventory; BRUMS: Brunel Mood Scale; CLBP: Chronic Lower Back Pain; CP: chronic pain syndromes; EHI: Edinburgh Handedness Inventory; EEG: Electroencephalogram; EMG: Electromyography; FIQ: Fibromyalgia Impact Questionnaire; FMS: Fibromyalgia Syndrome; HADS: Hospital Anxiety and Depression Scale; HAQ: Health Assessment Questionnaire; HP: Healthy Participants; HR: Heart Rate; HW: Healthy Women; PANAS: Positive and Negative Affect Schedule; PANAS-X: Positive and Negative Affect Schedule-Expanded Form; POMS: Profile of Mood States; PSWQ: Penn State Worry Questionnaire; SECS: Self-Expression and Control Scale; SF-12: 12-item Quality of Life Scale; SF-16: 16-item Quality of Life Scale; STAXI: State-Trait Anger Expression Inventory; STAXI-2: State-Trait Anger Expression Inventory; STAI: Spielberger State Anxiety Inventory; STAI-II: State-Trait Anger Inventory-II; RA: Rheumatoid Arthritis; TAS-20: Toronto Alexithymia Scale; VAS: Visual Analogue Scale; WHYMPI: West Haven-Yale Multidimensional Pain Inventory.

### 3.2. FMS and Anger

The current systematic review has an explorative nature and the selected studies included experimental tasks, descriptive studies, and interventions or treatments studies (such as mindfulness and strength training programs), among other designs to evaluate, treat (to reduce or to learn how to better cope with anger) or elicit an anger response, or analyze the attentional negative bias to anger.

#### 3.2.1. Anger in the Context of Personality Research

A predominant coping style of avoidance and anger, especially a high state anger and anger-in, was found in chronic pain patients [30]. In the same line, anger-in tend to be higher in FMS patients in comparison with healthy participants [24,27,28,29] and RA patients [27]. On purpose, anger-in and the anxiety scores foretold the level of pain severity in FMS patients [27]. However, it is interesting to note that in spite of anger-in is higher in patients who suffer from FMS, it is the behavioral expression of anger, along with anxiety, which predicts the pain severity in these patients [27].

Related to personality patterns, chronic patients seem to share some similar personality traits [30]. Several authors state that FMS patients do not significant differ from the other chronic pain patient groups (i.e., chronic lower back pain and RA patients) on personality traits [30]. In the case of anger as personality trait, anger seems not to be a characteristic personality trait in FMS patients [30].

#### 3.2.2. The Association between Anger and Other Relevant Variables in FMS

On the other hand, different researches studied the relationships between anger and clinical, emotional and/or physiological variables. In the physiological aspect, Shelley-Tremblay et al. [28] observed that FMS patients exhibited greater levels of depression and anger, among other indicators of distress as well as increased relative left-frontal activation (rLFA) than healthy participants. An association between anger and increased rLFA has been also suggested [28]. Furthermore, in a study conducted by González-Roldán et al. [41] FMS patients showed greater cardiac deceleration to all facial expressions compared to pain-free controls, and enhanced N100 amplitudes to pain and anger faces in comparison with neutral faces; supporting the presence of an attentional bias in FMS patients that would contribute to the automatic encoding of pain-related and unpleasant bodily information. Moreover, in the same study, a greater theta power was observed in response to pain and anger faces, along with more reduced alpha power than pain-free controls to all faces; pointing out that a higher recruitment of attentional resources and a more elaborated stimulus encoding seem to characterize the brain processing of pain and anger faces in FMS patients [41].

Regarding clinical, cognitive, and emotional variables, van Middendorp et al. [29] were the first authors to analyze both the general tendency to regulate anger (trait anger regulation) and its actual regulation in anger-arousing situations (state anger regulation). In this study, state anger predicted higher end-of-day pain in the almost half of the FMS patients (in 166 of 333 FMS patients), but lower pain in one-quarter of patients [29]. However, while state anger inhibition was unrelated to pain [29], trait anger inhibition was related to higher levels of pain [29]. Lower pain levels among FMS patients scoring high in trait anger expression (who actually expressed their anger in an anger-arousing situation) were also found [29].

Altogether, anger and sadness seem to amplify pain in women with and without FMS [44]. In fact, a stronger emotion-induced pain response was linked to more emotional reactivity [44]. Nevertheless, no conclusive evidence was found for a larger sensitivity to anger and sadness in female FMS patients than in women without FMS, or for a larger sensitivity to anger than to sadness in female FMS patients [44]. Likewise, anger and sadness were confirmed to be general risk factors for pain amplification [44], which emphasizes the need to implement emotion regulation techniques in clinical practice in order to reduce emotional pain sensitization in FMS patients [44]. 

Moreover, FMS patients exhibit higher levels of state and trait anxiety, worry, and angry rumination than other chronic pain patients (e.g., osteoporosis and osteoarthritis) [42,45] and healthy participants [42]. Simultaneously, worry and angry rumination has been strongly associated within the FMS group [42]. All anger rumination scales have been associated with a poor mental health and quality of life [45]. Moreover, the reported differences on mental health and quality of life between FMS patients and healthy participants have proposed to be mediated by anger rumination [45]. Additionally, in the study of Offenbaecher et al. [24], FMS showed higher pain and anger and poorer health and quality of life, which in turn were associated to lower levels of both, forgiveness of self and forgiveness of others [24].

It has also been observed that people with FMS who score high in alexithymia (difficulties in identifying and describing subjective feelings together with external-oriented thinking) attributed more pain to faces that express anger in facial recognition tasks compared to the rest of basic emotions [40].

#### 3.2.3. Interventions Aimed at Reducing or Better Coping Anger in FMS

Concerning physical activity, tension and anger showed a positive association with tests that require strength in knee extension in female FMS patients [43]. The mood states have been demonstrated to be also factors which may favor or impair motor performance [43]. El Tassa et al. [43] using the Profile of Mood States (POMS) instrument, authors reported that the greater success in physical performance is generally manifested by factors such as greater values of vigor (positive factors) and lower values of tension, depression, fatigue, confusion, and anger (usually associated with a physical depressed state). This study highlights a likely association among mood states (i.e., anger), depressive symptoms, and physical function (particularly physical function by field-based fitness tests). However, the lack of further studies at this regard should be accounted for.

Lastly, only two studies [46,47] have proposed and examined anger intervention programs. Amutio et al. [46] developed a mindfulness-based program intervention which resulted in a considerable decrease of anger (trait) levels (measured by the State-Trait Anger Expression Inventory), internal expression of anger, state anxiety, and depression together with an important increase in internal control of anger in the FMS compared to the FMS control group (waiting list) [46]. The mindfulness-based treatment was effective after seven weeks and its benefits were long lasting three months after the end of the intervention program [46]. Similarly, Andrade et al. [47] developed a strength training program to improve the mood state of FMS patients. The strength training program had a positive impact on the patients’ mood state after a single session [47]. Reductions in levels of anger (state), mental confusion, depression, fatigue, and tension due to the strength training program were also reported [47].

### 3.3. Quality of Selected Studies

C.M.G.-S. and C.I.M., performed a detailed evaluation of each study and any discrepancy was resolved by discussion with the rest of the authors. Due to the homogeneity of the selected manuscripts, the analysis was focus on the limitations of each study.

The main limitations of studies included the specification of two different diagnostic criteria for FMS in the same research [42,47] (for instance the 1990 and 2010 criteria [42] or the 1990 and 2016 criteria) [47]. In both studies [42,47] it is not clear on which diagnostic criteria the study based on. Although the 1990 ACR criteria was replaced by the 2010 criteria and nowadays new diagnostic proposals were undertaken (i.e., 2016 criteria), the major part of healthcare practitioners continue employing digital palpation or 1990 ACR criteria, in which is difficult to control the level of pressure exerted, and over or underdiagnosis seems to appear [18,19,48,49,50]. Additionally, it has been pointed out that the 1990 criteria and 2016 proposal entail different measures of FMS [51]. The 1990 criteria emphasize peripheral allodynia (tender points), whereas the 2016 emphasized the central pain perception and distress [51]. Therefore, it is important to inform about the used FMS diagnostic criteria. Other limitations found were: (1) the non-specification of the diagnostic criteria used at all [24,45]; (2) the non-report of the mean and standard deviation of the age of healthy participants [42]; (3) the low sample size (<20 participants) [28]; and (4) the non-clarification of how the sample size was calculated [24,27,28,29,30,40,41,43,44,46]. In fact, only three studies reported the procedure for the determination of the sample size [42,45,47]. The previous analysis also reveals the need to overcome the research gap referred to the variety of methodological designs. It would be recommendable to perform similar methodological studies to draw stronger conclusions. In addition, it is necessary to evaluate differentially anger as a state and trait in order to better know its characteristics. Unfortunately, only few studies measured state and trait anger [24,27,30,41,42,46].

As is pointed in the discussion, the reported limitations need to be overcome in future studies to better understand the relation between FMS and anger, establish the possible gaps of this research and optimize the FMS intervention.

## 4. Discussion

**Figure 2:**
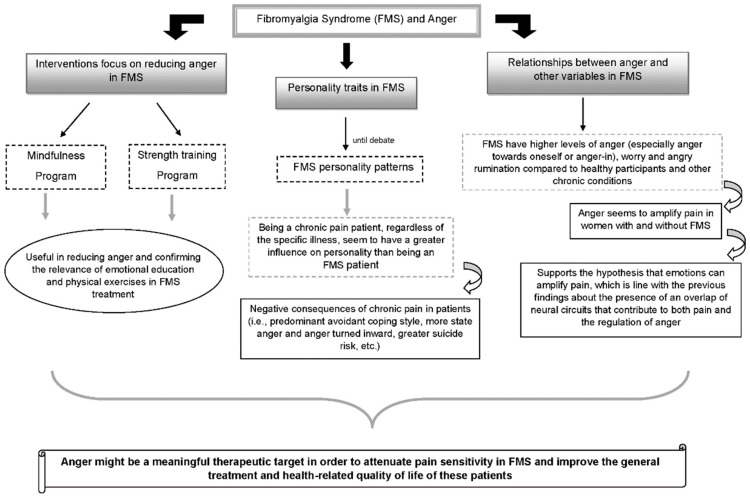
FMS and Anger.

The studies reviewed about anger have been heterogenous. To a better clarification and understanding, in the current discussion it will be presented the three main directions in this review: studies related to (1) personality traits in FMS, (2) the relationships between anger and other variables in FMS, and (3) interventions focused on reducing or managing anger in FMS (see Figure 2, elaborated by authors, for more detail and comprehension).

### 4.1. Anger in the Context of Personality Studies

Firstly, the question about whether exist or no a personality trait pattern in FMS remains under debate, and therefore its possible components, including anger, are also under debate.

The absence of an FMS personality pattern [30] has been explained through different mechanisms. Firstly, it is well-known that it may be not relevant to research about ‘‘permanent’’ personality traits since they are uncovered by the standard measures [52]. In addition, personality traits are always influenced or interact with the environment, and this interaction is required to be analyzed in personality research [52].

Given the previous ideas, some authors state that being a chronic pain patient, regardless of the specific chronic pain illness, seems to have a greater influence on personality [30]. It can be argued that suffering from a chronic illness might be more relevant than suffering specifically from a pain disease. It is likely that these personality characteristics are the result of the chronic condition and its coping [23]. Therefore, future research should also study the factors associated with the personality traits themself instead of the existence or non-existence of the traits per se.

In spite of not existing or being under debate the existence or not of a FMS personality patterns, it has been confirmed that the presence of chronic pain has negative consequences in patients (e.g., predominant avoidant coping style, more state anger and anger-in, greater suicide risk, etc.) [30].

In the case of anger, its presence in pain patients has been assumed since a few decades ago [53,54]. Specially, with regard to anger-in, some clinicians consider this as a normal reaction to not so regular situation [30]. The tendency of showing anger-in might be related to the fear of losing support from friends and relatives [27]. Moreover, anger-in could affect both, mental and physical health and contribute to reduction of health-related quality of life of these patients [55]. At the same time, it is possible that the behavioral expression of anger leads to increased pain sensitivity by provoking sympathetic activation [27]. Anger also can become chronic. In this sense, chronic angry emotional reactions are often maladaptive since they are associated with predominant chronic sympathetic activation and interpersonal disruption [55].

In conclusion, although, as indicated above, a pain-prone personality seems not to exist and the personality patterns reported in several research result from facing to a chronic stress situation such as chronic pain, chronic pain patients and healthy participants are supposed to significantly differ in personality, indicating that the specific disease seems not to determine the pattern since it depends on the chronicity of the same [30]. Nonetheless, it should be noted the relevance to include the emotional factors for better approaching aimed at getting better health outcomes in the diagnosis, treatment, and prevention of FMS [23,50,56].

### 4.2. The Relationships between Anger and Clinical, Emotional and Cognitive Variables in FMS

Secondly, referring to the relation between anger and clinical, emotional, and cognitive variables in FMS (i.e., clinical pain, anxiety, depression, etc.), the majority of the studies has focused on the level of anger per se more than in the associations between clinical, cognitive and emotional FMS variables and anger; or the impact of anger in FMS patients; which should not be overlooked as another important research gap.

Most of the analyzed studies in this review report that women [24,27,28,30] and men [24] with FMS have higher levels of anger (especially anger-in compared to healthy participants [24,27,28,29,30] and RA patients) [27]. Nonetheless, one study found no convincing evidence of increased sensitivity to anger in women with FMS [29].

Regarding the physiological aspects, frequently, researchers have analyzed the association between brain physiology and mood through power spectra electroencephalography (EEG) analyses, specifically within the alpha power band (8–12 Hz) [57]. Alpha waves are usually related to a wakeful relaxed state, particularly visible in occipital regions when the eyes are closed, whereas cortical deactivation seems to be linked to an increase in alpha amplitude in response to a specific stimulus or task [58]. Furthermore, lower relative right hemisphere compared to left hemisphere EEG oscillations, especially in the frontal lobes, is associated with behavioral approach-oriented emotions including anger and positive mood states [57]. Relatively greater right, in comparison with left frontal EEG asymmetry seems to be also associated with negative emotions (i.e., depression and anxiety) [28]. This indicates, as Harmon-jones [59] suggests, that the motivational direction component (approach/withdraw) is the key peculiarity that determines the relative left or right frontal activation, instead of the valence component (positive/negative).

Based on previous findings, Shelley-Tremblay et al. [28], showed a significant correlation between anger-hostility and rLFA -limited to the frontomedial sites- in FMS patients. Apart from these results, in the study of Shelley-Tremblay et al. [28] healthy and FMS participants exhibited a steadily increase in distress. Nevertheless, while the healthy participants reported the distress as a more withdrawal-related depression response, FMS patients experienced the distress as an approach-related, anger response. These results might be suggesting the possibility of a unique pattern of response to negative events in FMS patients [28], which need to be more deeply investigated.

Moreover, previous research indicate that a carbohydrate-face diet might improve chronic and idiopathic post-prandial mood symptoms in FMS patients [28]. On purpose, the Carbohydrate Addict’s Scale scores (a 17-binary forced-choice items questionnaire, to determine carbohydrate cravers, and therefore, the presence of a low carbohydrate diet), correlated with post-prandial rLFA in FMS [28]. This association is consistent with the showed appetitive approach-related motivation in the presence of negative affect in FMS patients [28]. FMS patients might ingest sweets to cope with negative physical and emotional feelings [28]. In fact, eating disorders, including greater levels of emotional eating, have been reported in FMS patients [60,61]. Based on the finding of a tiding between rLFA and positive reinforcement and anger [28], the ingestion of sweets by FMS patients may be self-reinforcing, yet tend to increase not only emotional but also physical symptoms [28]. This hypothesis needs to be consistently studied, since if it is confirmed, positive implications for FMS treatment recommendations might surface [28]. 

The research of Shelley-Tremblay [28] also found no evidence regarding a positive bolster in mood after consuming glucose in FMS patients. In fact, since 0 and 60 min to post-prandial high alpha oscillations arose. A possible explanation for self-medication with carbohydrates can be gain ability to be concentrated on immediate task demands, and not the improvement of mood itself [28]. Once again, our review indicates a research gap in this case of the effects of consumption of carbohydrates and glucose in FMS. In addition, in age-matched controls, the negative association observed between frontal alpha power and blood glucose may detail a decrease in frontal cortical activity associated to lessen blood glucose [28]. However, the lack of this association in FMS patients likely identify a cortical-associated alpha rhythm structures or processes disruption [28]. The presence of altered alpha, and its possible relation to thalamic abnormalities, is in line to several previous studies that stated the role of a thalamic dysfunction in FMS symptoms and etiology [62,63,64].

Psychophysiological responses to pain copying in chronic pain patients have also been studied [41]. It is well-known that suffering from FMS is related to a vulnerability to the effects of negative mood and a pattern of selective processing or cognitive attentional bias to the encoding of pain-related information [65,66,67]. In addition, it is further known that altered affective processing might be involved in the development and maintenance of pain and other affective symptoms related to FMS [20,68,69,70]. In this context, brain and cardiac activity elicited by viewing facial expressions of pain and anger in others is suggested to be impaired in FMS patients [41], showing an attentional negative bias which has even so far proposed as an assessment and treatment psychobiological marker in FMS. An increased mobilization of attention resources to pain and anger faces, along with a reduced allocation of attention to happy faces as well as an enhanced defensive reaction have characterized information processing in FMS [41,71].

Regarding psychological variables in FMS, behavioral expression of anger, along with anxiety, seems to be the best predictors of pain severity in FMS patients [27]. Furthermore, van Middendorp et al. [29] reported that state anger predicted higher end-of-day pain in half of the FMS patients, but lower pain in one-quarter of these patients. While state anger inhibition was not associated with pain in this study, trait anger inhibition was associated with higher pain [29]. Lower pain level among patients with high trait anger expression were also found, indicating that anger and a general leaning to inhibit anger predicts heightened pain in the everyday life of female FMS patients [29]. These results are in line with former research which demonstrated the pain-enhancing effects of anger-in, in anger induction experiments, mostly in persons who generally experience high levels of anger [12,34,72]. The current evidence suggests that the experience of both, anger and/or sadness amplify pain in women with and without FMS [44], which is congruent with the suggested presence of an overlap of neural circuits that are involved not only in pain but also in the regulation of anger [73]. As anger expression usually predicts less pain [29], the use of therapeutic emotional expression techniques in the treatment of clinical population including FMS may be beneficial [74,75,76]. 

Other negative emotions have been reported along with anger as a maladaptive distress copying style in FMS patients, for instance, rumination [42,45]. In this sense, rumination with anger has been significantly linked to poorer mental health and general health-related quality of life in patients with FMS [42,45]. In FMS patients, worry and rumination might be acting as coping strategies to deal with the negative emotional experience, although paradoxically an emotional well-being is not obtained [42]. The relationship between anger expression and health consequences is probably not linear. Ruminating and anger [77] and the mere expression of anger without cognitive processing [77] have been proposed to be maladaptive coping strategies themselves and might be mediating factor of the above relationship (see the Multiple Systems Model of Angry Rumination developed by Denson [78]). Altogether, these findings provide insights supporting the expression of one’s anger as a protector against rumination, as well as a factor in solving an emotionally problematic situation, lessen anger intensity and pain.

The relation between negative affect and daily pain in chronic pain patients has been well-established [79,80]. Negative emotions seem to be experienced with more intensity in FMS patients compared to the general population [20]. Accordingly, to the strong emotions neurophysiologic pain highlight, a greater associated pain processing in FMS is likely observed [72].

Di Tella et al. [40] reported that FMS patients who scored high in alexithymia attributed more pain to faces that express anger in facial recognition tasks. Alexithymia, instead of FMS per se, seems to play a relevant role in understanding the reported differences in pain attribution to anger-related facial expressions [40]. Anger processing might contribute to explain the specific significance attribution of pain to angry faces in FMS [40]. FMS patients usually exhibit high levels of alexithymia, a personality disposition affecting emotional self-awareness [50,56,71,81,82,83,84,85]. Alexithymia may be conceptualized as a cognitive style characterized by a problem in identifying and describing subjective feelings, and low externally oriented thinking [86,87]. Furthermore, the involvement of anterior cingulate cortex (ACC) in alexithymia has been suggested in several studies [88,89,90], reinforcing the idea that an altered ACC activity compromised functioning in persons who experience high levels of alexithymia [91]. Alexithymia is associated with a delayed preparation to process biologically prepotent events as measured by the orienting complex (N2/P3a); delayed preparation is even stronger for angry faces [91]. Based on the fact that ACC is considered as the principal source of these neurophysiological components, these results are in line with the hypothesis concerning the impairment in ACC functioning in alexithymia by indexing its delayed contribution around 300 ms [91]. Empirical evidence also supports ACC reactivity is positively associated with the anger intensity [92]. More ACC activation was reported when the face depicted anger with a greater intensity [91].

Another research framework is related to forgiveness in chronic pain in general and FMS in particular. Forgiveness has been linked to pain, anger, and psychological distress in chronic pain patients [93]. Forgiving oneself and others may be included in the psychosocial care of FMS patients to potentiate enhanced mental health and quality of life and reduced anger [24]. In this sense, FMS patients usually exhibited lower levels of forgiveness not only of self but also of others [24]. The inclusion of forgiveness as a therapeutic target may facilitate FMS patients use it as a coping strategy to better deal with symptoms (both psychological and physical), and improve the general well-being [24]. Forgiveness, which is considered both a state and a trait, is a multidimensional construct that refers to oneself or others [94]. In the field of well-being promotion studies, the trait forgiveness (in face of its momentary state) is of a considerable interest due to its influence over time and situations and general greater positive psychological impact [95]. In healthy participants, forgiveness has been associated with a variety of psychological well-being indicators such as reduced anger, along with lower depression and anxiety [24,96,97,98]. Forgiveness has been also related to reduced sympathetic arousal, enhanced parasympathetic tone, and physical health and longevity [96,97,98]. In addition, in several studies using a heterogeneous sample of pain patients with various etiologies, locations, and pain duration and extension, forgiveness has been related to less time spending in avoiding or fighting again pain, reduced pain intensity and pain interference, as well as greater levels of mental health [99]. It is necessary to promote protective psychological mechanisms such as forgiveness in the wake of facilitate its potential to relieve symptoms of pain and psychological distress. This may reduce anger, promote emotional well-being, and improve quality of life in FMS patients [24]. Without detriment to the above, more studies are necessary to evaluate the anger intervention, as a state, since as a trait it is difficult to modify.

### 4.3. Interventions Focus on Reducing or Managing Anger in FMS

Although the findings reviewed support the undoubted clinical relevance of anger in FMS, only two studies [46,47] have up to date applied a treatment for it. Amutio et al. [46] observed that mindfulness was effective in reducing anger (trait) in FMS patients. Authors conclude that due to the prevalence of negative emotions in FMS and the problems to manage them [28,29,65,66,67], mindfulness seems to be a good therapeutic strategy for managing negative emotions. Mindfulness leads chronic pain patients to experience awareness and acceptance of the sensations and feelings related to their symptoms whereas they continue physically and mentally active and focus on their daily life and values [46]. Mindfulness can be conceptualized as being focus on experiences in the present moment [100]. Two basic components of mindfulness are self-regulation of attention and acceptance of one’s own experiences in a non-evaluative way (in other words, a non-reactive awareness) [101]. In consequence, mindfulness seems to be a potent pathway to learn and apply new non-reactive models of responding to the emotional suffering and tolerate pain (without suffering) associated with different disorders, including FMS [46]. However, more studies are required to document the effectiveness of mindfulness programs in the reduction and management of anger in FMS patients.

Regarding physical activity in FMS patients, tension and anger showed a positive correlation with tests that demand strength in knee extension [43]. In addition, stressful interpersonal events [102], general negative affect [103], and anger in daily life [44] increase pain sensitivity and the reactivity of pain-relevant physiologic and muscle tension [36,104]. Nevertheless, more research is required to investigate whether a possible negative mood state (i.e., depressive, with predominance of anger, etc.) in the chronic pain population, influences the accomplishment of certain physical tasks (i.e., walking, running, etc.) or even tasks that reflect the activities of the daily life (i.e., doing housework, shopping, etc.) [43]. The findings of El Tassa et al. [43] clearly reveal that some components of mood states (i.e., tension and anger) are associated with the physical function of women with FMS. However, this is still a research line little explored [43]. On purpose, to relieve the physical and psychological symptoms and, in consequence, the general health of FMS patients, it is well-known that physical exercise is proposed as a valid alternative treatment since past years [47,105,106,107,108]. Between the several types of physical exercise, the most effective treatment option to reduce common symptoms of FMS has been strength training [109,110,111,112,113,114]. In the clinical trial performed by Andrade et al. [47] a reduction was reported in anger levels in FMS by a strength training program. Although these results demonstrate acute health positive outcomes to physical exercise in FMS patients [47], as it occurs with the above-mentioned study [46], more research is still needed to confirm these conclusions.

The previous results support that FMS patients who express their anger have a better clinical profile (i.e., lower level of clinical pain) [42,44]. It has also been confirmed that the psychological intervention could be also focus on developing a healthy expression of anger to reduce its negative impact on FMS patients [42,44,46,115]. It is important to note that FMS treatment guidelines emphasize the importance of patient psychoeducation and self-care strategies for the management of symptoms, and advise for the need of integrating non-pharmacologic with pharmacologic treatments [116]. Based on previous studies, both reducing anger and expressing it in a proper and healthy way seems to be useful in FMS treatment. In special, it is advisable to conduct more studies to evaluate whether specific anger reduction improves different clinical variables (i.e., a moderation analysis to check what effect has anger on the effectiveness of treatment in the rest of the clinical parameters) and whether is better express and/or reduce anger. Up to date, it seems that both (reduction and proper anger expression) are positive for FMS patients, but the remain question is if the effects of both entail equal benefits. Nevertheless, as it has been become aware along this review, more studies on the subject, in general, are needed to draw stronger conclusions. Although the majority of analyzed studies have been focus on assessing anger instead of treat it, and considering the relation between anger and the rest of aforementioned explained variables (i.e., pain, anxiety, depression, alexithymia, attentional bias, low forgiveness, altered psychophysiological responses, low health-related quality of life, etc.), it is recommendable to develop and evaluate treatment programs to instruct patients about how manage anger and, as a result, relieve their symptoms.

To summarize, this review points out some important future lines of research, such as, the circumstances in which certain personality traits are elicited rather than the existence or non-existence of the traits per se; the pattern of reactions to negative events in FMS patients; the presence of emotional eating disorders in FMS patients; the relation of anger reduction with general and positive health parameters instead of negative variables; the expression, management and levels of anger in male FMS patient; the consumption of carbohydrates and glucose in FMS, and its effects; and the possible associations among mood states (i.e., anger), physical function (particularly with reference to physical function by field-based fitness tests), etc. Regarding treatment, it is necessary to design more treatments and to develop research to assess if specific anger reduction improves different clinical parameters (i.e., anxiety, depression, pain, fatigue, insomnia, rumination, worry, coping strategies, health-related quality of life, etc.). 

## 5. Conclusions

To the best of our knowledge, this systematic review is the first to be conducted on the relationship between FMS and anger. Anger-in tends to be higher in FMS patients compared to healthy participants and RA patients. FMS patients also exhibit greater levels of state and trait anxiety, worry and angry rumination than other chronic pain patients. Anger seems to amplify pain in women in general, especially those with FMS and affects more the health-related quality of life of FMS patients. In spite of the relevance of emotions in the treatment of chronic pain, including FMS, only two studies have proposed intervention programs focus on anger treatment, indicating a positive reduction in anger levels through mindfulness and a strength training program, respectively. Additionally, this systematic review highlights some important research gaps, as well as the need to study anger and state anger in general more than focus on anger due its overall temporal and situational consistency. Considering the influence of negative emotions on chronic pain (for example, anger, sadness, etc.) it is vital that anger is studied more deeply in FMS in order to provide insights directed to improve the diagnosis, treatment, and quality of life of these patients. More research is also needed on the anger subject in men with FMS since gender differences (i.e., in coping strategies, symptoms, treatment adherence, etc.) are relevant in chronic disease and treatment need to be personalized. In addition, FMS patients are likely to present with anger in clinical practice. Therefore, skills for handling with angry patient are essential for primary care providers at all levels.
